# Beyond the Workplace: How Psychological Empowerment Influences Employee Creativity in the Saudi Hotel Sector—The Mediating Role of Affective Commitment and the Moderating Role of Psychological Safety

**DOI:** 10.3390/ejihpe15050076

**Published:** 2025-05-09

**Authors:** Ahmed Mohamed Hasanein, Musaddag Elrayah

**Affiliations:** Management Department, College of Business Administration, King Faisal University, Al-Ahsaa 380, Saudi Arabia; melrayah@kfu.edu.sa

**Keywords:** workplace, psychological empowerment, employee creativity, hotel sector, affective commitment, psychological safety

## Abstract

Businesses in the hotel and hospitality sectors face significant challenges when they lack originality and creativity. Creativity and innovative behavior are both necessary to increase employee effectiveness. This study aims to investigate how psychological empowerment (PEMP) directly affects employee creativity (EC) and affective commitment (AC) in the Saudi hotel industry. Additionally, this study examines the moderating function of psychological safety (PS) and the mediating role of affective commitment. Data were collected through an online cross-sectional survey of a purposive sample of 536 operational staff at hotels in the Eastern Region of Saudi Arabia. Structural equation modeling was used to test the proposed framework (PLS-SEM). Surprisingly, these findings revealed no significant direct effect of PEMP on EC. However, AC has emerged as a crucial mediating mechanism through which PEMP indirectly fosters creativity. Furthermore, psychological safety (PS) is found to moderate the PEMP–EC relationship, enhancing its impact under favorable conditions. These results highlight the theoretical and practical significance of emotional commitment as a conduit for empowering employees to engage in creative behavior. This study offers new insights into how psychological and organizational factors interact to shape creativity in the hospitality context.

## 1. Introduction

Currently, many businesses aim to gain a competitive edge. The hotel industry is undergoing rapid changes, especially in the wake of global crises. An environment of mobility, constant shifts, and economic and technical complexity now permeates the tourism industry, challenging organizations to adapt and innovate (Breier et al., 2021; Gössling et al., 2020; Teo et al., 2025).

Hospitality and hotel businesses that lack originality and inventiveness find it difficult to thrive in the market. Creativity and inventive behavior are essential components to develop employee performance (Nasifoglu Elidemir et al., 2020). By encouraging employees’ inventive conduct and giving them more authority, managers can positively impact both individual and enterprise creativity (Çekmecelioğlu & Özbağ, 2016). Individuals are more inclined to participate in creative initiatives when companies promote innovative behaviors and supply the required tools (Çekmecelioğlu & Özbağ, 2016). Material and intellectual organizational capital, which includes human resources, are considered firm assets, according to many researchers (Barney, 1991). Unlike easily replicated tangible assets, businesses need to cultivate their human capital and acquire irreplaceable, diverse, and intuitive human capital. Traditional beliefs about human creativity are being reshaped by the growing digitization of many facets of life and work. Academics and professionals have raised many questions about how to encourage staff innovation in the setting of a digital workplace (Cai et al., 2020).

Numerous researchers from various new fields, including artificial intelligence, have given the problem of creativity and its immediate effects a great deal of attention in the literature today (Runco, 2025). Creativity is beneficial for organizational success (Oldham & Cummings, 1996). In previous years, many researchers have studied the direct impact of creativity in the workplace. Studies have found that employees’ intrinsic motivation, domain-relevant skills, and creativity-relevant capacities can all be improved by workplace wellness; consequently, employee creativity increases (R. Miao & Cao, 2019). Creativity, or the application of innovative ideas, is among the most crucial components of competitiveness in organizations in the twenty-first century (Kremer et al., 2019).

Creativity is a key goal for prosperous modern businesses. However, although creativity is highly valued, it is difficult to achieve in practice. Currently, the majority of businesses and organizations understand the importance of creativity (Slåtten et al., 2020). According to the study findings, management is the most potent predictor and moderator of staff creativity (Bavik & Kuo, 2022). Previous findings have demonstrated that a favorable environment and corporate culture have a significant impact on worker creativity (Bavik & Kuo, 2022).

Drawing from the self-determination theory (SDT) (Gagné & Deci, 2005) and creativity theory (Kozbelt et al., 2010), this study aims to determine how psychological empowerment (PEMP) influences employee creativity in the Saudi hotel sector. The degree to which workers feel capable, think the job is fulfilling, and are committed to their employment is known as psychological empowerment, which is a four-dimensional characteristic that is seen as an essential workplace resource (Spreitzer, 1995).

By removing all obstacles that promote helplessness through formal business procedures and informal means of sharing practical knowledge, psychological empowerment provides employees with a sense of esteem (Conger & Kanungo, 1988). In addition, this study aims to explore the mediating role of affective commitment and the moderating role of psychological safety. Knowledge dissemination and job participation were favorably and strongly linked with empowered management (Joo et al., 2023).

By thoroughly examining the direct influence of PEMP on employee creativity (EC) in the Saudi hotel business, our study aims to close a theoretical gap in comparison to prior studies in the hotel sector. Additionally, our study contributes to the existing body of literature by providing a thorough understanding of the role of affective commitment (AC) in mediating this link. Furthermore, we add to the corpus of previous research by investigating the moderating function of psychological safety (PS). Previous studies have not tested these relationships. Therefore, our model is unique in that it examines the mediating role of affective commitment and the moderating influence of psychological safety, both of which have not been modeled before. The primary research question is how psychological empowerment affects employee creativity. Additionally, this research attempts to address the following questions: Does affective commitment (AC) act as a mediator in this relationship? Does psychological safety (PS) modify this relationship?

## 2. Literature Review

### 2.1. Psychological Empowerment and Employee Creativity

Contact with coworkers is a source of people’s psychologically experienced empowerment, which in turn leads to an increase in the personal drive to act professionally and with purpose (Orgambídez-Ramos & Borrego-Alés, 2014). It is a mental state that is connected to how perceptions evolve at work and motivates people to operate to the best of their abilities for the benefit of the organization, using four dimensions of empowerment (Huey Yiing & Zaman Bin Ahmad, 2009). The conceptual frameworks that will direct the development of our hypotheses are provided by the social exchange theory (SET) (Blau, 1964) and the self-determination theory (SDT) (Deci & Ryan, 2000).

According to the standard of return (Gouldner, 1960) and social exchange theory, workers who believe that the enterprise will benefit them are more likely to feel compelled to return the favor and become more positively engaged in their work. Individuals who are made aware that their enterprise views them as important assets to the company’s survival and achievement are often more empowered (García-Chas et al., 2014). Previous investigations show that creative thinking and psychological empowerment (PEMP) are directly related (Younas et al., 2023). It is essential to discuss PEMP to understand employees’ perceptions of their roles. Four cognitions—meaning, competency, freedom of choice, and impact—are the foundation of this perspective (Ali et al., 2020).

Several significant findings were produced from research on psychological empowerment, organizational isolation, and innovative behavior among Saudi Ministry of Sports personnel. Both organizational deviance and innovative behavior were found to be significantly correlated with psychological empowerment. This suggests that workers’ innovative behaviors and connections with enterprise objectives strengthen as they feel greater autonomy (Alibrahim, 2024). 

Greater psychological empowerment rates among employees have been shown to foster their capacity for creativity, which is essential for enterprise creativity (Liu et al., 2019; Singh & Sarkar, 2012; Siyal et al., 2023). Prior research has revealed a strong correlation between innovative behavior, organizational variations, and psychological empowerment (Alibrahim, 2024).

According to a different study, psychological empowerment positively influences worker creativity, both directly and indirectly, through the mediation effects of motivational factors and creative procedure involvement (Nguyen & Doan, 2023). Another study discovered that while psychological empowerment has a major impact on creativity via motivational factors, it has no immediate impact on creativity among staff members (Hirmawan et al., 2023). Therefore, we postulate the following:

**H1.**  *Psychological empowerment in the workplace has a positive effect on employee creativity*.

### 2.2. Psychological Empowerment and Affective Commitment

In organizational contexts, psychological empowerment is frequently thought to influence a variety of behaviors, including creative behavior (Javed et al., 2019; Seibert et al., 2011). Affective commitment is directly and favorably impacted by psychological empowerment, as this study shows. According to the study findings, for example, staff members are more inclined to form more emotional ties with their enterprise and exhibit greater affective commitment when they consider themselves inspired (Yogalakshmi & Suganthi, 2020). Additionally, there is evidence that psychological empowerment can improve workers’ evaluations of their abilities (Ruiz-Fernández et al., 2022). We argue that this evaluation depends on workers’ experience, qualifications, and satisfaction at work.

Studies have shown that psychological empowerment has a substantial beneficial effect on affective commitment. This emphasizes the importance of providing workers with the tools they need to strengthen their psychological bonds with the enterprise, which will eventually result in increased enthusiasm and involvement in the workplace (Diniyati & Sudarma, 2018). Researchers discovered a strong correlation between affective commitment and psychological empowerment, suggesting that workers with greater degrees of psychological empowerment are also more likely to have better degrees of affective commitment, which will eventually boost their level of happiness at work (Aggarwal et al., 2024). Based on these discussions, the following hypothesis is proposed:

**H2.**  *Psychological empowerment in the workplace has a positive effect on affective commitment*.

### 2.3. Affective Commitment and Employee Creativity

Organizational commitment, according to Meyer et al. (2002), is a behavior that unifies a person’s relationship with the organization to make the aims of the organization and individual align. Prior research has demonstrated that staff with lower levels of organizational commitment are more likely to commit errors at work (Lambert et al., 2013), feel more anxious (Glazer & Kruse, 2008), exhibit greater disagreements with their families, and avoid additional duties than those with higher levels of organizational commitment. Different studies have found that people with a connection to an organization generate inventive concepts and respond better to difficulties (Amabile, 1988). Additionally, dedicated workers and satisfied workers are able to overcome obstacles and accomplish their objectives using less money (Licata et al., 2003). The relationship between leadership qualities and employee creativity is mediated by fulfillment through work (Semedo et al., 2016). Employees can act creatively when they are happy with their positions. Research indicates that job satisfaction mediates the association between employee creativity and supervisor openness, underscoring the significance of an encouraging work environment in promoting creativity (S. Miao et al., 2020).

According to a previous study, employee creativity is greatly influenced by affective commitment. This emphasizes how affective commitment, which, when combined with creative job thinking, boosts creativity, is fostered by real leadership (Semedo et al., 2016). Thus, by encouraging employee creativity, true management may indirectly improve organizational effectiveness by promoting affective commitment. Another study showed that individuals’ innovative behavior is greatly influenced by their affective commitment to a variety of goals, including assignment and field of employment. This highlights that controlling employee creativity is essential for innovative outcomes in organizations, even though it does not specifically address work satisfaction or organizational performance (Van Rossenberg, 2014). Hence, we propose the following hypothesis:

**H3.**  *Affective commitment in the workplace has a positive effect on employee creativity*.

### 2.4. The Role of Affective Commitment and Psychological Safety Between Psychological Empowerment and Employee Creativity

A staff member’s sense of connection and affiliation with an organization is reflected in their affective commitment, a crucial aspect of organizational commitment that influences their willingness to stay and make a meaningful contribution (Eby et al., 1999). Social exchange theory is one of the main psychological processes underlying affective commitment. When workers believe that the enterprise values them, encourages them, and gives them resources to advance, they become more affectively committed (Li et al., 2022). By encouraging sentiments of option, independence, self-worth, and meaningfulness, psychological empowerment mediates the association between job quality and affective commitment. This results in enhanced commitment, as workers believe that their work has an impact and fulfills them (Pentareddy & Suganthi, 2015). According to a prior study, nurses’ job involvement and empathy leadership are mediated by affective organizational commitment. These psychological processes are thought to improve work behaviors by creating a sense of intent and personal connections to the organization (Zhang et al., 2024). Furthermore, the association between participatory leadership and organizational citizenship behavior is strongly moderated by ongoing commitments (Hayat Bhatti et al., 2019).

The degree to which employees of an organization feel at ease and are willing to take risks together is known as psychological safety. It is defined by social respect and confidence (Edmondson, 1999). When employees develop connections with others at work that are reliable and constructive, they feel more psychologically protected (Men et al., 2020). Previous findings suggest that followers who exhibit great psychological stability are less likely to be afraid of taking risks, such as the possibility of suffering injury or adverse effects, while expressing their thoughts or creativity (Jin et al., 2022). Therefore, the following hypotheses were proposed:

**H4.**  *AC mediates the relationship between PEMP and EC*.

**H5.**  *PS moderates the interaction of PEMP on EC*.

**Figure 1:**
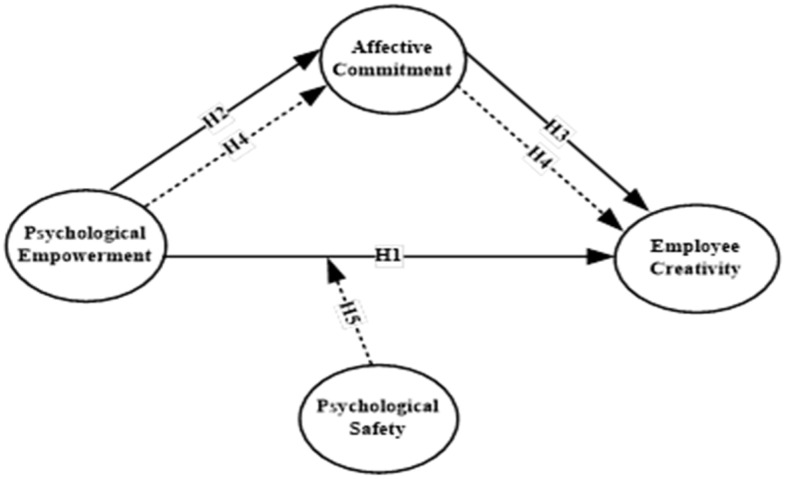
Research Conceptual Framework.

The conceptual framework used in our study is displayed in Figure 1 below:

## 3. Methods

This study examined the relationship between psychological empowerment (PEMP) and employee creativity (EC) in the Saudi hotel industry, focusing on the mediating function of affective commitment (AC) and the moderating interaction of psychological safety (PS). A theoretical framework was developed based on previous research. The data from the online survey aided in the validity assessment of the model. Based on the statistical techniques of probable least squares structural equation analysis (PLS-SEM) and starting as a concept with bias (CMB), a comprehensive survey was designed to meet the research objectives. A relatively condensed summary of the research methodological technique is given in the following sections.

### 3.1. Sampling and Data Collection

A purposive sampling method was used to select Saudi Arabian hotel employees from the eastern region. An online cross-sectional survey was conducted between January and March 2025. The survey design and administration followed Hair et al.’s (2006) methodological guidelines. The survey instrument was initially reviewed via a pilot prior to distribution to determine its clarity and accuracy. We disseminated the survey link via email and across different social media platforms to ensure maximum outreach to the actively employed hotel workers in the region. Data reliability was maintained by continuously monitoring responses during collection. The researchers also offered contact information to address any participant concerns in the introduction to the survey.

To ensure that ethical research standards were upheld, all participants were provided with full knowledge of how the study would be run before participating. Before the survey, they were assured of their anonymity, and they provided written consent. Through both professional and personal networks, participants were recruited to act as intermediaries. Additionally, all respondents were assured that participation in the data collection was for an explicit research purpose and everyone’s presence was voluntary.

### 3.2. Research Instruments

Five distinct parts comprised the study survey, all of which were carefully designed to obtain certain critical constructs of the study. The demographic background of the primary respondents was gathered for the first section, which comprised age, gender, education, and tenure, to provide a contextual background for analysis. The second section used a 12-item scale in the development of psychological empowerment (PEMP) as measured by Spreitzer (1995). The PEMP scale has been shown to have high internal consistency (Cronbach’s alpha = 0.980). In the third section, employee creativity (EC) was assessed using a 6-item scale developed by Tierney et al. (1999). This scale was also found to be strongly internally consistent based on reliability analysis with a Cronbach’s alpha of 0.959.

The fourth section used a 6-item scale for affective commitment (AC) developed by Meyer and Allen (1991). Including this scale measures the employees’ emotional attachment to the hotel. A Cronbach’s alpha of 0.951 confirmed the reliability of the scale in the current study. The fifth section deals with psychological safety (PS) using a 7-item scale adapted from Edmondson (1999). This study also showed excellent reliability for the scale, with strong consistency of Cronbach’s alpha (0.955). Using a seven-point Likert scale, participants’ responses were recorded, where “strongly disagree” was scored one (1) and “strongly agree” was scored seven (7). In order to determine content validity, the entire survey was reviewed by 11 academic experts in the hospitality sector, including for wording, industry expressions, and clarity of the questions. The experts did not suggest revisions prior to the survey being distributed.

### 3.3. Data Evaluation

The study was conducted with a total of 750 full-time hotel employees, who were selected as participants and received the corresponding questionnaires. A response rate of 71.5% (536 completed responses) with no missing data was achieved upon completion of the validity checks. According to Nunnally and Bernstein’s (1994) suggestion to have at least 10 items for each respondent, the minimum sample size required in this study was 394, which was successfully met. Out of the 536 responses that were taken as valid, 461 (86%) were male and 75 (14%) were female. Furthermore, most of the respondents (81.5%) were aged 26 to 35 years. Most participants (84.5%) were working in either the front office or food and beverage departments.

Descriptive statistics and multiple regression analyses were performed using Smart PLS-V 4.01 for data analysis, and additional statistical procedures were performed using SPSS version 24, which was based on the two-stage approach proposed by Leguina (2015). The partial least squares structural equation modeling (PLS-SEM), as proposed by Henseler et al. (2009), was the most suitable method for both exploratory and predictive research in accordance with the criteria as described by Henseler et al. (2009). Moreover, Hair et al. (2017) and do Valle and Assaker (2016) suggest that the sample size is sufficient to permit distributional flexibility in the normality assumption. CMV was addressed through Harman’s single-factor test implementation in line with the recommendations by Podsakoff et al. (2003).

## 4. Results

Exploratory factor analysis (EFA) provides preliminary evidence of the multidimensional structure and validity of the measurement model. According to an exploratory factor analysis (EFA) of the 27 items, the first factor only accounts for 21% of the variance. Although the data setup is naturally multidimensional, this outcome highlights the fact that the first element is responsible for a considerable amount of variance. This suggests that additional latent variables may be needed to account for item relationships. There seem to be a few issues with the common method variance (CMV) in the current study. Moreover, variance inflation factor (VIF) values below the 5 threshold (Table 1) demonstrate the lack of multicollinearity. According to Table 1, each scale item’s standardized factor loadings (λ) are above the 0.7 criterion, indicating strong convergent validity and consistent measurement of the corresponding construct.

The measurement model demonstrates robust discriminant validity based on established psychometric criteria. According to the recommendations of Fornell and Larcker (1981) and Hair et al. (2017), the proposed model’s ability to explain the variance of the components that the variables are linked to is better than that of the other constructs, as demonstrated by the relevance between the variables and their corresponding components (see Table 2). This confirms the model’s discriminant validity. In addition, the study reveals that the factor loading for every item is stronger on its related concept than on any other variable, thereby supporting the model’s discriminant validity according to Chin (1998).

In order to determine discriminant validity, the Heterotrait–Monotrait (HTMT) ratio is used to assess the degree of similarity between latent constructs; according to Henseler et al. (2016), an HTMT score below 0.90 will confirm discriminant validity; as Table 3 illustrates, all HTMT ratios fall below this 0.90 threshold, thereby supporting robust discriminant validity. Because the constructs in the model are demonstrated to be separate concepts that do not significantly correlate, this finding aids in enhancing the reliability of the results.

The structural model offers nuanced insights into the relationships between psychological empowerment (PEMP), affective commitment (AC), psychological safety (PS), and employee creativity (EC) in the Saudi hotel sector. Table 4 presents the results that shed light on the effects of psychological empowerment (PEMP) on employee creativity (EC) in the Saudi hotel sector in terms of the mediating effect of affective commitment (AC) and the moderating effect of psychological safety (PS). The results indicate that hypothesis (1) is rejected because the direct relationship between PEMP and EC (H1) (β = 0.062, T-value = 0.933, *p*-value = 0.349) is not statistically significant. It implies that the role of PEMP may be to work on the development of employees’ creative capacities, but it does not directly translate into the creation of more creativity in the workplace. This non-significant result suggests that other mechanisms, such as affective commitment or working conditions, may be needed to translate empowerment into creative outcomes.

**Figure 2:**
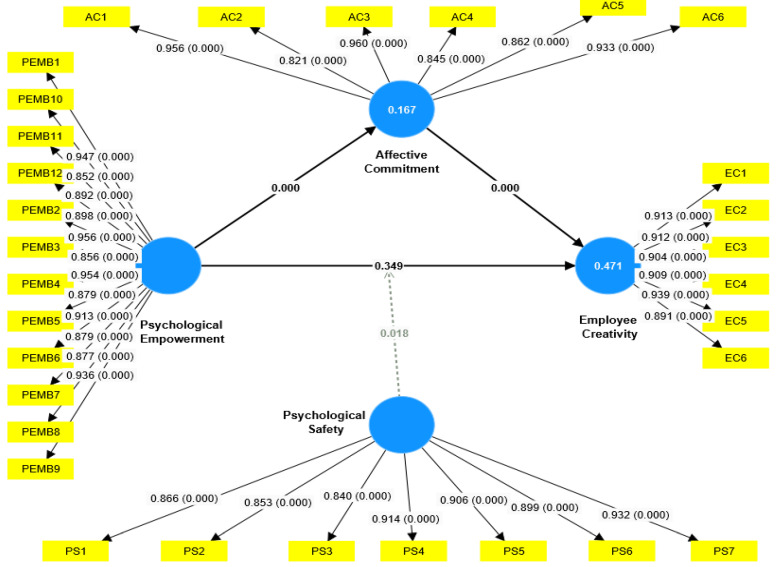
Final Research Model.

The results, however, strongly support the second hypothesis (H2) that PEMP positively affects AC (See Table 4 and Figure 2). This showed that there is a significant effect (β = 0.408, T-value = 6.367, *p*-value = 0.000 ***) and that employees who feel more empowered will be more likely to develop a powerful emotional attachment and commitment towards their organization. This finding reinforces the necessity to create an empowering work environment, not only to increase employees’ intrinsic motivation but also to stimulate employees’ emotional attachment to the work, thereby contributing to greater job satisfaction and performance. The third hypothesis (H3) explores the relationship between AC and EC and shows a strong and statistically significant effect (β = 0.568, T-value = 6.600, *p*-value = 0.000 ***). The result implies that employees who are more emotionally committed to their organization also show higher levels of creativity. This then leads us to believe that emotionally attached employees are usually inclined to put effort into contributing creative ideas and solutions in their workplace. AC proved to be a very significant positive influence on creativity, signifying the importance of emotional engagement to aid in creativity in the hospitality industry.

**Figure 3:**
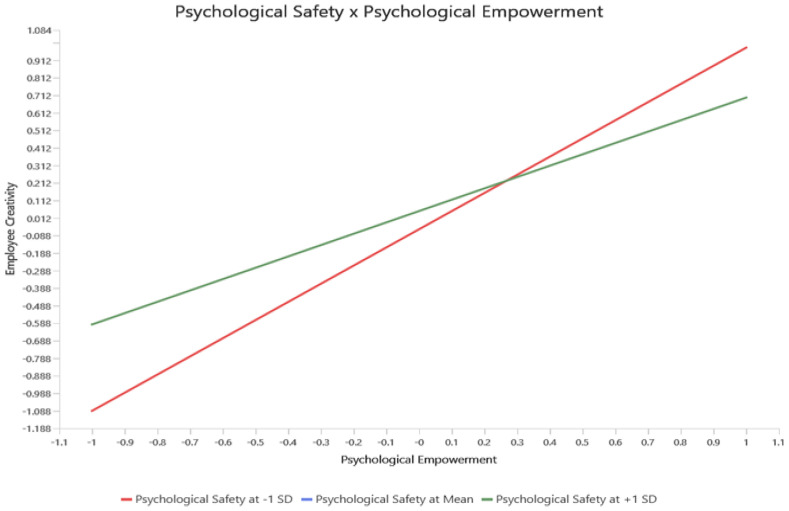
The Moderation Effect.

The indirect relationships between variables validate the mediating role of AC and the moderating effect of PS between PEMP and EC (see Table 4). With regard to the mediation analysis, hypothesis (4) further substantiates the role of AC in the relationship between PEMP and EC. The results reveal that AC fully mediates the relationship between PEMP and EC (β = 0.232, T-value = 4.309, *p*-value = 0.000 ***), confirming hypothesis (4), and demonstrate that PEMP affects EC indirectly through AC (H4), which acts as a critical mediating factor in the Saudi hotel sector. This suggests the possibility that although PEMP would not improve creativity directly, it could do so indirectly by positively enhancing employee affective commitment toward the organization. The mediation effect of this suggests that empowerment and commitment must be cultivated simultaneously by hotel managers to promote a more creative workforce. Also, the moderation analysis shows that the relationship between PEMP and EC is moderated by PS (H5) (See Table 4 and Figure 3). Results for the hypothesis support the fact that PS has a strong moderation interaction on the influence of PEMP and EC (β = 0.091, T-value = 2.362, *p*-value = 0.018 *). This reveals that PS impacts the effect of psychological empowerment on creativity (see Figure 3). Thus, the perception of employees’ psychological safety would result in them being more inclined to translate their feelings of empowerment into creative behaviors. In other words, when employees feel safe to voice their thoughts without worrying about the repercussions, they are more predisposed to embrace creative risks, which implies that PS is a key decision factor in maximizing employees’ empowerment potential toward creative behavior.

## 5. Discussion and Conclusions

This quantitative study aims to ascertain how employee creativity (EC) and psychological empowerment (PEMP) relate to one another. The purpose of the study is to ascertain how affective commitment mediates the link between PEMP and EC. In addition, this study tries to explore the moderating role played by psychological safety (PS) in the relationship between PEMP and EC. The current study employed a cross-sectional design, which limits the ability to draw causal conclusions. While the statistical models suggest associations and potential pathways of influence, the temporal ordering of variables cannot be definitively established. Therefore, any interpretations implying causality should be viewed with caution. Future studies employing longitudinal or experimental designs would be better suited to confirm the directional nature of these relationships and to provide a more rigorous test of the proposed causal mechanisms.

According to our results, the direct correlation between PEMP and EC is not statistically significant. It suggests that while PEMP may help individuals enhance their creative abilities, this does not necessarily result in increased workplace creativity. This non-significant finding raises the possibility that additional mechanisms, like affective commitment or working conditions, may be required to convert empowerment into innovative results. This result does not align with previous studies (Aswinaryanto et al., n.d.; Nguyen & Doan, 2023; Singh & Sarkar, 2012; Tierney et al., 1999).

Furthermore, this study shows that PEMP has a favorable impact on AC. This provides a significant indication that employees who experience greater empowerment are more likely to form a strong emotional bond and devote themselves to their enterprise. This result highlights the need to establish an empowering workplace in order to boost employees’ inner drive and emotional investment in their work, which will ultimately lead to improved satisfaction with employment and output. This result supports previous research findings (Diniyati & Sudarma, 2018; Yogalakshmi & Suganthi, 2020). The study investigates the relationship between AC and EC and found a statistically significant and obvious impact. According to the analysis, employees who are more emotionally invested in their organization are also more innovative. This lends credence to the idea that emotionally invested employees are generally more inclined to devote effort in order to develop innovative ideas and solutions for their jobs. AC significantly improved creativity, suggesting that emotional involvement is essential for promoting creativity in the hospitality industry. Our results are consistent with earlier research in the same area (Semedo et al., 2016; Van Rossenberg, 2014).

Furthermore, we investigate how AC contributes to the link between PEMP and EC with respect to the mediation study. Our findings show that AC completely mediates the relationship between PEMP and EC and that PEMP indirectly influences EC through AC, a crucial mediating element in the Saudi hotel industry. This result raises the potential that, despite not directly fostering creativity, PEMP may indirectly foster it by strengthening employees’ emotional attachment to the enterprise. According to the mediation effect, hotel managers should foster commitment and empowerment at the same time to facilitate a more innovative workforce. This unique result adds value and contributes to the current literature.

Lastly, our moderation study reveals that PS moderates the link between PEMP and EC. The impact of psychological empowerment on creativity is revealed by PS. Employees who feel psychologically safe are therefore more likely to translate their sense of empowerment into innovative actions. To put it another way, employees are more likely to take creative risks when they feel free to express their opinions without fear of the consequences. This suggests that PS is a crucial determinant in optimizing employees’ empowerment and capacity for innovative behavior. This unique result also contributes to the current literature.

## 6. Implications and Recommendations

### 6.1. Theoretical Implications

This study contributes theoretical insights that expand conceptual boundaries of employee creativity (EC) and psychological empowerment (PEMP) by applying these constructs to the Saudi hotel industry. The study applies the self-determination theory (SDT) to show how PEMP creates three intrinsic elements of autonomy and competence alongside relatedness that boost employee creative capabilities. This study enhances research knowledge through its demonstration of how supported employees tend to form deep emotional connections with their organization, which drives greater creative participation. PS plays a moderating role as it demonstrates the need for workplaces to create environments where staff can present innovative thoughts securely without facing unfavorable feedback. The comprehensive research model demonstrates that creativity within Saudi Arabian service industries stems from the interactive forces linking psychological empowerment to affective commitment under an organization’s established cultural norms. Leadership strategies can benefit from these discoveries to develop creativity-enabled empowerment systems that focus on psychological wellness outcomes.

### 6.2. Practical Implications

This research provides important practical applications that guide hotel managers and hospitality leaders to improve employee creativity through psychological empowerment. First, hotel managers should establish PEMP as a management priority due to its ability to enhance employee intrinsic motivation, together with autonomy and competence. Hotel managers should empower their workforce by both offering employees control over their decisions and developing their required skills while supporting their sense of ownership of their responsibilities. A transition toward empowerment-driven leadership in the Saudi hotel industry with its conventional hierarchies will create a more engaged workforce that innovates and solves creative problems in delivering services to customers.

Second, PEMP strengthens affective commitment, which mediates the relationship between PEMP and employee creativity (EC). However, those employees with an emotional connection to the organization will be more likely to have higher levels of commitment and creativity. Commitment is fostered when hotel leaders develop a strong organizational culture that celebrates employee contributions, gives meaningful recognition, and emphasizes how employee goals fit into the overall company vision. A range of initiatives, such as mentorship programs, team-building activities, and incentive-based rewards, can serve as powerful mechanisms for fostering emotional engagement and sustaining employees’ creative momentum.

Third, the role of empowered employees’ psychological safety (PS) moderation, the degree to which they are confident in expressing their ideas, must also be considered. Even the most empowered employees are hesitant to take creative risks in a psychologically unsafe work environment. Open communication, constructive feedback, and non-punitive mistakes should be encouraged by hotel managers, who should also make the employees comfortable with sharing innovative ideas. The hospitality leaders in Saudi Arabia can unlock their employees’ creative potential and facilitate better service quality, customer experience, and competitive advantage by embedding psychological safety into workplace culture.

Fourth, hotel managers should pay attention to the balance between autonomy and support, which is important for the creation of both psychological empowerment and creative potential from the employees. It is important to encourage employees to make decisions while also providing them with the guidance and resources to succeed. To accomplish this, managers can provide ongoing training and access to tools that help create the desired creativity while ensuring all employees know the overall goals and expectations of the organization. The balance between supporting employees’ freedom and making sure they have the support they will need to be successful will help employees to take the initiative, yet feel confident they have the backup they need to be successful, which will improve both their creativity and performance.

Finally, given the socio-cultural context of the setting, empowerment strategies should be implemented with sensitivity to both organizational and cultural dynamics. In societies characterized by high power distance and collectivist orientations, such as Saudi Arabia, employees may not instinctively translate feelings of empowerment into creative behavior unless additional psychological conditions are met. Our findings underscore that empowerment alone does not significantly predict creativity; rather, its influence is conditionally mediated by affective commitment and moderated by psychological safety.

Therefore, hotel leaders should design empowerment initiatives that not only resonate with local values but also intentionally foster emotional attachment to the organization and cultivate a climate of psychological safety. This may involve culturally tailored incentives, adaptive leadership approaches that encourage inclusive participation, and mechanisms that signal the organization’s shared commitment to collective success. By embedding empowerment within a supportive and emotionally engaging work environment, hotel managers can more effectively enable the latent creative potential of their employees to emerge in ways that are meaningful within the cultural context.

## 7. Study Limitations and Future Directions

This research provides valuable insights about how psychological factors affect employee creativity in Saudi hotels, yet it contains several limitations. The cross-sectional research design prevents researchers from establishing cause-and-effect relationships. The application of structural equation modeling (PLS-SEM) in statistical testing produces advanced results, yet fails to determine which variables preceded others in time. Future research must use both longitudinal designs and experimental approaches to prove directional flow between the variables seen in this study.

Second, the research design incorporates self-reported data, which potentially introduces common method bias, yet statistical analyses indicate such a bias remains at small levels. Future research analyzing emotional intelligence in service workers needs to address this limitation either by combining ratings from different sources or waiting for a sufficient time after each evaluation.

Third, the sample is geographically and contextually bounded, comprising hotel employees from the eastern region of Saudi Arabia. Although this setting offers novel insights into an under-researched context, the findings may not generalize to other regions or industries. Future research should explore whether similar mediating and moderating dynamics exist across different national cultures, sectors, or job roles.

Finally, the investigation of affective commitment and psychological safety found successful results as mediators while also working as moderators, yet alternative structural aspects, such as organizational support, job autonomy, and leadership styles, could influence the psychological empowerment–creativity relationship. The theoretical framework would achieve greater completeness as an understanding of organizational creativity through the inclusion of additional variables.

## Figures and Tables

**Table 1:** Measurements and variable parametric attributes.

Scale Variables	λ	VIF
Psychological Empowerment: (α = 0.980, CR = 0.982, AVE = 0.817)		
PEMP1	0.947	1.504
PEMP2	0.956	1.464
PEMP3	0.856	1.419
PEMP4	0.954	1.921
PEMP5	0.879	1.273
PEMP6	0.913	2.565
PEMP7	0.879	1.506
PEMP8	0.877	1.495
PEMP9	0.936	2.637
PEMP10	0.852	2.214
PEMP11	0.892	2.691
PEMP12	0.898	1.537
Psychological Safety: (α = 0.955, CR = 0.963, AVE = 0.788)		
PS1	0.866	2.313
PS2	0.853	2.023
PS3	0.840	1.404
PS4	0.914	1.182
PS5	0.906	2.493
PS6	0.899	1.864
PS7	0.932	2.186
Affective Commitment: (α = 0.951, CR = 0.961, AVE = 0.807)		
AC1	0.956	2.564
AC2	0.821	1.791
AC3	0.960	1.723
AC4	0.845	2.693
AC5	0.862	2.713
AC6	0.933	1.464
Employee Creativity: (α = 0.959, CR = 0.967, AVE = 0.831)		
EC1	0.913	2.814
EC2	0.912	2.041
EC3	0.904	1.973
EC4	0.909	2.943
EC5	0.939	2.963
EC6	0.891	1.714

**Table 2:** Assessing discriminant validity using the Fornell–Larcker criterion.

	AC	EC	PEMP	PS
AC	**0.898**			
EC	0.664	**0.911**		
PEMP	0.408	0.259	**0.904**	
PS	0.916	0.642	0.581	**0.888**

Note: Bold figures show the square root of AVE; HTMT ratios are shown in brackets.

**Table 3:** Assessing discriminant validity using the Heterotrait–Monotrait ratio.

	AC	EC	PEMP	PS
AC				
EC	0.691			
PEMP	0.413	0.262		
PS	0.892	0.614	0.799	

**Table 4:** Hypotheses testing results.

	β	T-Value	*p*-Values
**Direct Effects**			
(H1) PEMP → EC	0.062	0.933	0.349
(H2) PEMP → AC	0.408	6.367	0.000 ***
(H3) AC → EC	0.568	6.600	0.000 ***
**Indirect Effects**			
(H4) PEMP → AC → EC	0.232	4.309	0.000 ***
(H5) PS X PEMP → EC	0.091	2.362	0.018 *

Note: [*** *p* < 0.001; * *p* < 0.05].

## Data Availability

Data are available upon request from researchers who meet the eligibility criteria. Kindly contact the first author privately through e-mail.
